# Impact of the Empathic Understanding of People and Type D Personality as the Correlates of Social Skills of Primary Health Care Nurses: A Cross-Sectional Study

**DOI:** 10.3390/ijerph20010201

**Published:** 2022-12-23

**Authors:** Agnieszka Chrzan-Rodak, Grzegorz Józef Nowicki, Daria Schneider-Matyka, Elżbieta Grochans, Barbara Ślusarska

**Affiliations:** 1 Department of Family and Geriatric Nursing, Faculty of Health Sciences, Medical University of Lublin, Staszica 6 Street, 20-081 Lublin, Poland; 2 Department of Nursing, Pomeranian Medical University in Szczecin, Żołnierska 48, 71-210 Szczecin, Poland

**Keywords:** nursing competences, primary health care, social skills, empathy, type D personality, personality traits

## Abstract

Efficient functioning at work depends on social skills. The aim of this study was to assess the relationship among empathy, type D personality traits, and the level of social skills among Primary Health Care (PHC) nurses. A cross-sectional study was carried out involving 446 PHC nurses. In the multidimensional model, after taking into account disruptive variables (age, place of residence, postgraduate education and self-assessment of health), as in one-dimensional models, respondents with a higher level of empathic understanding of other people were found to have a higher level of social skills (b = 0.76; SE = 0.11; *p* < 0.001), while a lower level of social skills was observed among respondents with traits which are characteristic of type D personality (b = −11.86; SE = 2.28; *p* < 0.001). The results of the study show that personal predispositions, such as empathy or type D personality, may support or hinder the shaping of social skills of nurses. Therefore, it is essential to create an individualised approach when nurses are undergoing social skills training.

## 1. Introduction

Nurses in Primary Health Care (PHC) provide safe and effective disease prevention, diagnosis, treatment and rehabilitation care. The core competences for nurses in PHC include patient education, effective communication, teamwork and leadership, people-centered care and clinical practice, and long-term learning and research [1]. The European e-Delphi survey identified 28 core competences for family and community nurses, providing a practical and concrete summary of the needs of local populations through a continuous process based on the prevention of illness and the promotion of health, safety and self-care education [2]. In addition to education, prevention of illness, health promotion and nursing care, PHC nurses also play a role in facilitating contact and interactions among patients, social and health services and hospitals, as well as acting as advocates for the needs of individuals and their families [3].

Contemporary analytical approaches understand the structure and development of basic nursing competences as being based on the concept of holism, which sees competences as a set of elements which are required in specific professional contexts, e.g., knowledge, skills, attitudes, thinking ability and values [3]. Identifying the measurable components of nursing competences through the concept of holism and integration poses a challenge. Many researchers have tried to identify the main elements by using various definitional and structural models [4,5,6]. While performing their profession, nurses integrate all their skills and use them to ensure effective patient care [7].

**Figure 1:**
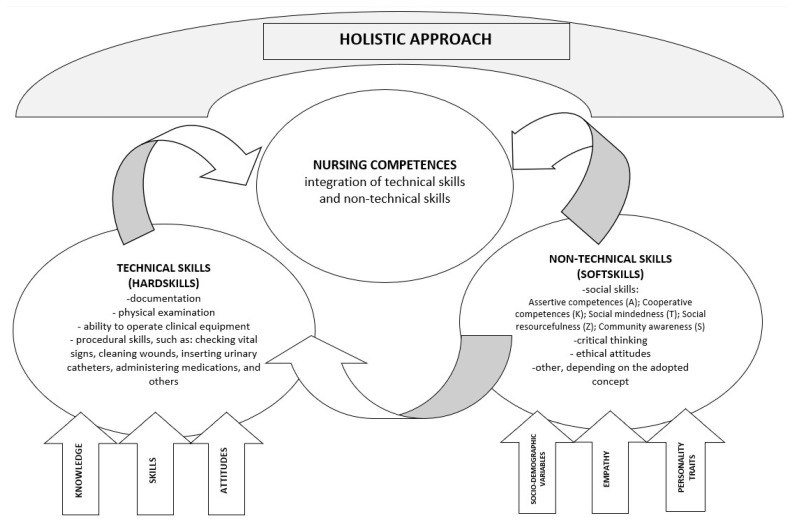
Conceptual model: A holistic approach to nursing competences.

In a holistic approach, a nurse integrates technical and non-technical skills. Technical skills are motor skills involving the use of medical equipment such as needles, syringes, test tubes, infusion pumps, catheters, probes, etc. These seemingly simple and mechanical skills require complex movements, theoretical knowledge as well as critical thinking skills [8]. The second type of skills are non-technical skills, also known as soft skills, which, although described differently in different settings, may include social skills, critical thinking or ethical attitudes [7,9,10,11,12]. Soft skills function as an independent component conditioning a holistic approach to nursing competences while simultaneously affecting the quality of technical skills [13]. A model presenting a holistic approach to nursing competences is presented in Figure 1 [7,8,9,10,11,12,13].

Among non-technical skills, social skills deserve special attention, due to their broad basis. Social skills include learned behaviours that make interactions with others more manageable, i.e., allowing an individual to function efficiently in a social and professional environment [14]. Social skills include verbal and non-verbal communication, problem-solving styles (e.g., rational, impulsive and avoidant), self-confidence, assertiveness, goal setting and self-control [15,16,17,18]. According to Semrud-Clikeman, social skills integrate several elements, including emotional, cognitive and behavioural ones [19]. Due to their various uses depending on the situation, those elements ensure the social adaptability of the individual and their ability to function effectively in social settings [15,20]. Additionally, enhancing social skills is essential in personal and professional development, as this aids in building better relationships with others [21].

Nurses’ social skills are linked to their personal and professional self-esteem, which influences their ability to engage in effective communication with patients and improve care outcomes [22]. The results of many studies confirm that the relationship between nurses and their patients, based on the social skills of the former, clearly has an impact on the well-being and of the latter. A high level of social skills is more likely to result in a better quality of care and more satisfaction with it, which is of particular importance for PHC patients [13,23,24,25,26].

Social skills also have positive aspects for the nurse. Studies have demonstrated that people with better social skills enjoy higher levels of social support and a better quality of life and are resistant to depressive disorders [23,27]. Thus, strengthening the practices that require social skills among health professionals and nurses is essential to improve the satisfaction and quality of care provided [23,24,25]. To achieve this goal, the focus should be placed on learning and developing the components of interpersonal relationships requiring social skills.

Empathy is of particular importance in effective patient-focused communication [28]. Empathy, understood as the ability to feel and understand the states of others, is one of the most important social skills. It is also one of the communication tools we use to understand others and share our feelings, thoughts and experiences. Empathy is the ability to feel another person’s emotions, feelings and thoughts and to position oneself in a way that can reinforce effective patient-focused communication, especially in the context of improving problem-solving skills [29]. Empathy can be explained both emotionally and cognitively. Emotional empathy means feeling another person’s emotions and giving the most appropriate response based on their emotional state. This response is very important in patient–nurse interactions. Cognitive empathy is the ability to recognise another person’s feelings without experiencing them yourself [30]. Therefore, empathy strengthens social skills and enables people to communicate, learn, ask for help, meet needs in the right way, get along with others, make friends, develop healthy relationships and protect and generally interact with society.

In contrast to the factors strengthening social skills, there are certain personality traits that can cause a decrease in the level of social skills. An example of such traits may be those related to personality type D. Personality type D, called the “stress” personality, is a specific type of personality characterised by two dimensions: negative emotionality and social withdrawal. Individuals with this type of personality tend to experience negative emotions—depression, anxiety, anger or hostility. They perceive themselves negatively and report many complaints about somatic ailments. In addition, they show a tendency for social withdrawal, a unique style of interacting with others which is intended to avoid any potential threats from social interactions, such as, for example, a lack of approval from others. Such people often feel uncomfortable, tense and have no sense of security when they meet others [31,32]. The D-type personality construct is based on a relatively permanent disposition which manifests through two main dimensions of personality—neuroticism and low extraversion [32].

The complex structure of social skills and their integrative nature in professional contexts create difficulties in fully understanding them [16]. Currently, the healthcare system requires nurses to fully use their professional potential, including their social skills—with the diversity of their determinants—to solve patients’ problems and needs flexibly and creatively [1,2]. However, there is a knowledge gap regarding the determinants of social skills among nurses, especially PHC nurses. Determinants such as empathic understanding of other people and the traits characterising type D personality play a unique role in shaping the quality of contact and interactions with patients and their families [2,6]. Therefore, the aim of our work was to assess the relationship between empathy and type D personality and the level of social skills among PHC nurses.

## 2. Materials and Methods

The results presented in this article are a part of a large project whose aim is to assess the factors influencing the social skills of PHC nurses. Some of the results, including basic correlations, are presented in the publication “Empathy and Type D personality as correlates of the level of social skills of Primary Health Care nurses [33].

### 2.1. Study Design

**Figure 2:**
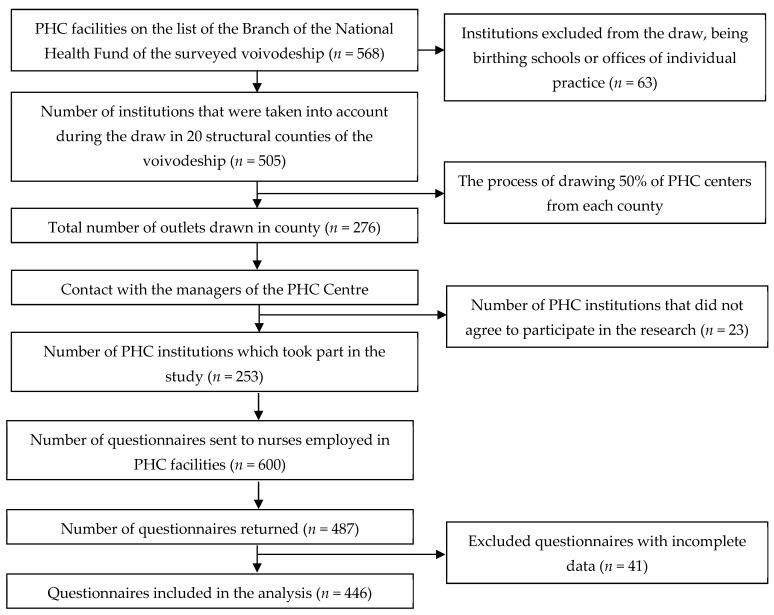
Procedure for collecting PHC Centres and respondents’ forms.

Cross-sectional studies involving primary health care nurses were conducted in 2019/2020 using a diagnostic survey method in the form of paper/pencil research material collection (PAPI). The research was conducted in randomly selected PHC Centres located in the Lubelskie Voivodeship in eastern Poland. The sample selection was made in two stages (Figure 2). Stage I: a random selection of PHC Centres. In the first stage, from the website of the National Health Fund [34], we collected a list of PHC Centres operating in the selected province. Sixty-three institutions that were individual practices or prenatal schools were excluded from the list. The selection included 505 PHC Centres from 20 counties divided according to structural affiliation in the voivodeship. We replaced the specific names of the institutions with an assigned number. The large number of facilities throughout the voivodeship led to the inclusion of half of the available facilities. Simultaneously, we considered that the sample we chose should, in the best way, reflect the surveyed population. Tickets with numbers from each county were placed in 20 containers, and then 50% of the PHC Centres were randomly selected. In total, 253 PHC Centres were drawn from all 20 counties. Then, the institutions were contacted by phone and permission was sought from the relevant managers to include the nurses employed in those centres in the research. In case of refusal, another facility from the same county was selected, such that half of the PHC facilities from each county participated in the research. Managers who consented to participate in the study received questionnaires to be distributed to nurses in that PHC facility. Stage II: a cross-sectional survey of PHC nurses. A total of 600 questionnaires were emailed to the enrolled facilities. Four hundred eighty-seven completed surveys were returned (81.16% return rate). The criteria for inclusion of respondents in the analysis included: expression of informed consent to participate in the research, at least 6 months of experience in the nursing profession and correct completion of the questionnaires. The exclusion criteria were the opposites. After a thorough analysis, 446 surveys were included in the study.

The study was reported in the Strengthening the Reporting of Observational Studies in Epidemiology Studies (Table S1) [35].

### 2.2. Ethics Approval

Ethical approval was issued by the Bioethics Committee at the Medical University of Lublin (decision number: KE-0254/224/2018). The study was conducted in accordance with the principles of the Declaration of Helsinki. Each respondent received written information concerning their anonymous and voluntary participation in the research and each gave written informed consent to participate in the study.

### 2.3. Questionnaires

In order to achieve the aims of the study, a structured questionnaire was developed consisting of three standardised questionnaires and an author’s questionnaire. All scales used in the study complied with internal consistency standards (Table S2).

#### 2.3.1. Assessment of Social Skills

The PROKOS Questionnaire (The symbols used to designate scale and subscales in the PROKOS questionnaire are derived from their original Polish names. We have left the abbreviations of the original symbols, because there is no English version of this tool, and the authors of the questionnaire have not yet published the names of these subscales in a language other than the original one)—Social Competences Profile (Social Competences Profile) by Matczak and Martowska [12] was used to assess social competences. It is a self-description questionnaire consisting of 90 items, of which 60 are diagnostic items (on social competences) and 30 are buffer items (on non-social competences). Each item defines activities and tasks. The respondent’s task is to answer on a four-point Likert scale (from 1, “definitely bad”, to 4, “definitely good”) with regard to how they cope in a given situation. In formulating the individual items in the questionnaire, the authors considered situations and activities occurring in the lives of adults in five areas: their professional, social and family life, as well as their social activities and how they deal with everyday matters. An analysis of the results allowed us to calculate the overall result and the results for the individual subscales. The overall score constitutes a sum of points scored on all five scales, ranging from 60 to 240 points. According to the interpretation proposed by the authors, both the overall score and individual subscales score can be described as high, average and low, depending on the number of points obtained. The number of possible points varies across subscales:

- Assertive competences subscale (A—14 items)—this assesses each respondent’s ability to influence other people and the ability to refuse or object. The score in the subscale ranged from 14 to 56 points.
- Cooperative competences subscale (K—16 items)—this assesses the skills of establishing cooperation and resolving interpersonal conflicts. The score in the subscale ranged from 16 to 64 points.
- Social mindedness subscale (T—11 items) assesses the ability to establish and maintain social relationships. The score in the subscale ranged from 11 to 44 points.
- Social resourcefulness subscale (Z—13 items)—this assesses the ability to enforce one’s rights and privileges and influence others in achieving one’s goals. The score in the subscale ranged from 13 to 52 points.
- Subscale community awareness (S—6 items)—this assesses the ability to initiate and implement social goals, as well as engage others in these goals. The score ranged from 6 to 24 points.

#### 2.3.2. Empathy Level Assessment

The Empathic Understanding Questionnaire (KRE—We use the original abbreviation of the KRE scale, because there is no English version of this tool, and in the literature, the authors often use the abbreviation KRE) by Węgliński was employed to assess affective, cognitive and motivational empathy [36]. The tool consists of 33 statements to which the participants respond using a four-point scale, comprising “yes”, “rather yes”, “rather no” and “no”, referring to their thoughts and experiences related to interactions with other people. To calculate the results, a four-point rating scale was used for each statement of the inventory, using conversion factors according to formula A or B, from 3 points to 0 points. The tool includes questions concerning five empathetic tendencies: sensitivity to the experiences of others, emotional syntonies, being moved by the positive and negative emotions of others, empathising with the experiences of others and the willingness to make sacrifices for others; these are related to the overall result and allow one to assess the level of empathy of a given individual. Using the four-point scale, the respondent determines how they would behave in the situation described in the question, which requires a demonstration of empathy. The scale score is between 0 and 99 points, whereby a higher score indicates a higher level of empathy.

#### 2.3.3. Severity of Type D Personality Traits

The severity of Type D personality traits was assessed using the Type D Personality Scale (DS-14) by Denolett [37] in the Polish adaptation of Ogińska-Bulik and Juczyński [32]. The tool includes 14 statements to which participants respond using a five-point Likert scale (from 0—false to 4—true). Type D personality consists of two subscales: negative affectivity (NA), i.e., the tendency to experience emotions such as anger, anxiety and irritation, and subscale social inhibition (SI), i.e., isolation from society and refraining from expressing emotions. Seven of the 14 theorems define the NA dimension and seven determine the SI dimension. Obtaining a minimum of 10 points in a given dimension is the basis for qualifying the subject to a given subscale. Obtaining a minimum of 10 points in both subscales supports the diagnosis of a Type D Personality.

#### 2.3.4. Sociodemographic Variables

In the next set of questions, the respondents were asked to provide several sociodemographic variables necessary for the statistical development of the collected material. The questions asked were about age, gender, marital status, place of residence, education, whether the respondent completed some form of postgraduate education, whether the respondent had completed any forms of postgraduate education in the last two years and whether the respondent has additional work on top of what they already do in their basic place of employment. In the final question, the respondents were asked to self-assess their health condition on a Likert scale from 1—bad to 5—excellent. Research shows that certain personal factors, such as health condition, can be an important variable in determining the competence of nurses [38,39].

### 2.4. Statistical Analyses

The analysis was performed using the IBM SPSS Statistics for Windows, Version 26.0. (2019. Armonk, NY, USA: IBM Corp.). Data are expressed as means and standard deviation (SD) for numerical variables. The Shapiro-Wilk test was used to assess conformity with a normal distribution. The continuous variables were compared between groups using Student’s *t*-test (ANOVA—at least three groups). Categorical variables were analysed using the χ2 test. A Pearson correlation was used to assess the relationships between numerical variables. Multiple linear regression was used to assess the relationships between type D personality trait severity, empathy level and social skills. The variables significantly associated with social skills in univariable analysis were included in the multivariable model. The model was created after adjustments for age, place of residence, postgraduate education and perceived health were made. The results of the linear regression model are presented as a coefficient (b) with standard error (SE), and coefficient of determination (R^2^) was used to describe the appropriateness of performed model. The obtained results of the analysis were assumed to be statistically significant at *p* < 0.05.

## 3. Results

### 3.1. Characteristics of Participants

The study involved 446 nurses working in PHC. The mean age of the participants was 47 ± 10.19 years, and mean years in the profession was 24 ± 11.53 years. Respondents were predominantly female (89.91%; *n* = 401), in relationships (83.63%; *n* = 373) and living in a city with a population above 100,000 (46.19%; *n* = 206). As many as 87.44% (*n* = 390) stated that they had completed some form of postgraduate education during their professional career, amongst whom 67.04% had done so in the last two years. Among the forms of vocational training, nurses most often completed a qualification course (77.81%; *n* = 305). Additionally, 31.39% (*n* = 140) have a second job. The mean self-assessment health status was 3.08 ± 0.69. Detailed data on the sociodemographic characteristics of those surveyed are presented in Table S3.

### 3.2. Distribution of Analysed Characteristics According to PROKOS, KRE and DS-14

Table 1 shows the average scores of respondents on the scales used in the study. The average social skills score amongst the surveyed nurses was 172.27 ± 24.52. Amongst all the subscales, the highest mean score was observed in subscale (S), i.e., Community awareness, while the lowest was observed for subscale (K), i.e., Cooperative competences. The average empathy score of the PHC nurses in the Empathic Understanding Questionnaire was 64.63 ± 10.16. Finally, 172 nurses were characterised by having a Type D personality.

### 3.3. Relationship between Empathic Understanding Questionnaire, Type D Personality and Social Competences

**Figure 3:**
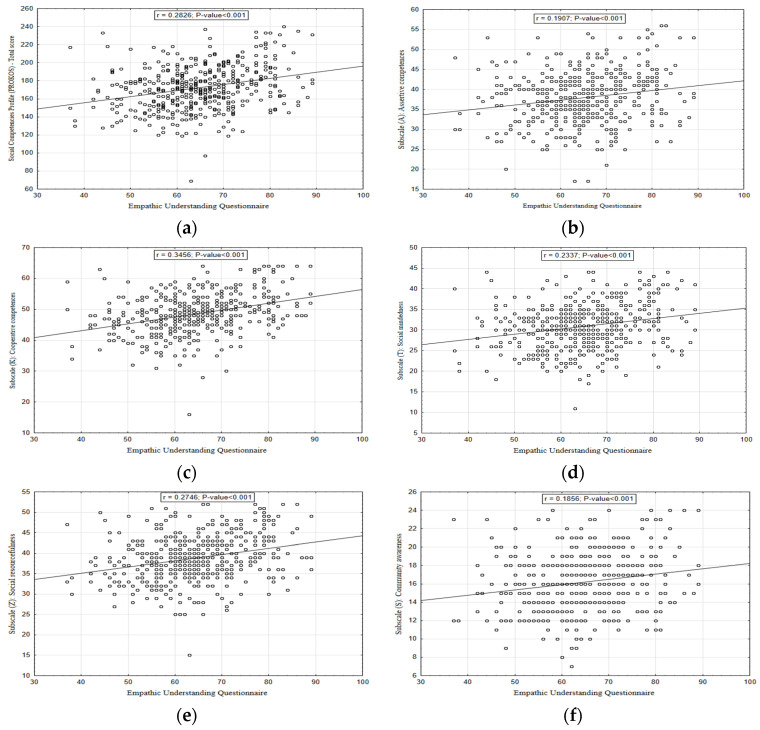
Relationships between Social Competences Profile and Empathic Understanding Questionnaire: (**a**) Social Competences Profile (PROKOS): Total score; (**b**) Subscale (A): Assertive competences; (**c**) Subscale (K): Cooperative competences; (**d**) Subscale (T): Social mindedness; (**e**) Subscale (Z): Social resourcefulness; (**f**) Subscale (S): Community awareness.

**Figure 4:**
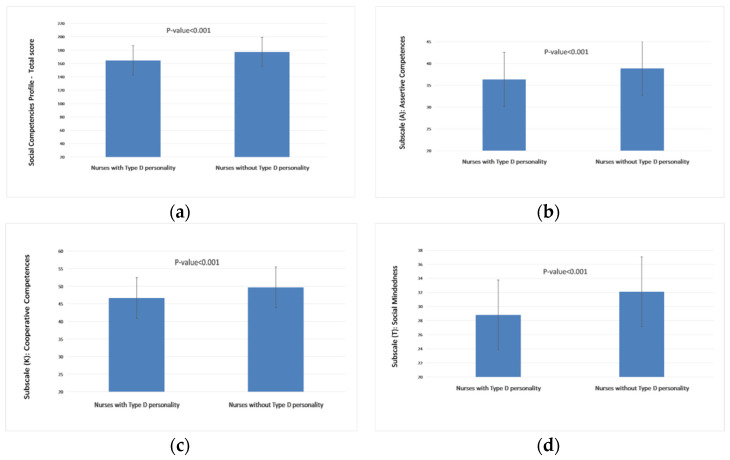

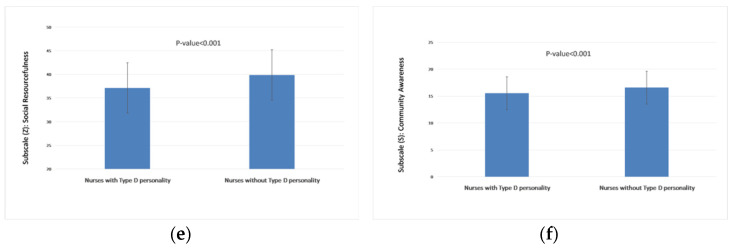
Relationships between Social Competences Profile and type D personality: (**a**) Social Competences Profile (PROKOS): Total score; (**b**) Subscale (A): Assertive competences; (**c**) Subscale (K): Cooperative competences; (**d**) Subscale (T): Social mindedness; (**e**) Subscale (Z): Social resourcefulness; (**f**) Subscale (S): Community awareness.

Figure 3 shows the relationships between the Empathic Understanding Questionnaire and the level of social skills. All subscales and the total social skills score were significantly and positively associated with the Empathic Understanding Questionnaire. The strongest relationships were observed in the case of Subscale (K): Cooperative competences. Figure 4 shows the relationships between a type D personality and the level of social skills. Participants with a D type personality had lower mean values on each subscale and total score.

### 3.4. Multivariable Analysis between Empathic Understanding Questionnaire, Type D Personality and Social Competences

Table S4 presents the relationships between selected sociodemographic features and level of social competence. Features significantly related to PROKOS in one-dimensional models were included in multidimensional models.

Table 2 presents the multivariable models describing the relationships between type D personality, the Empathic Understanding Questionnaire and level of social skills. After adjustments were made for age, place of residence, postgraduate education and perceived health, nurses with type D personality had significantly lower mean values on each subscale and total score for social skills. The differences in means values of PROKOS obtained in the regression model were similar to those in the univariable analysis. As in the univariate analysis, after taking into account the influence of potential confounders, the Empathic Understanding Questionnaire was significantly associated with all subscales and the total social skills score. The direction and strength of the investigated relationships were like those obtained in the simple analysis. The models with all variables are presented in Supplementary Materials Tables S5 and S6. A greater increase in the coefficient of determination was observed in the Empathy level assessment when the models with covariates and after adding this variable were compared (more than three-fold increase in the case of Subscale K).

## 4. Discussion

PHC nurses play an important role in the healthcare system, and their competences are a resource that strengthens the healthcare system. The professional skills of nurses are very complex and constitute a combination of knowledge, technical abilities, attitude, critical thinking and social skills [40]. Social skills are an important component of nurses’ competences which are influenced by many different factors, including external factors, but also personal predispositions [41,42]. The aim of our work was to assess the relationship between the empathic understanding of people and type D personality and the level of social competence among PHC nurses. In our research, most nurses were characterised by an average level of social competence. Among the examined PHC nurses, positive and significant correlations were observed between the empathic understanding of other people and the level of social skills. On the other hand, the relationship between a type D personality and social skills was significantly negative. In multidimensional analyses, disruptive factors were taken into account: age, place of residence, postgraduate education and self-assessment of health condition. An empathic understanding was a significant predictor of social skills due to the overall PROKOS scale and each of its subscales. In turn, type D personality was a predictor of lower results in the field of social skills in the overall result and each of the PROKOS subscales.

Personality traits play an important role in shaping competences. They can contribute to perseverance in establishing and improving competences by shaping a positive attitude towards their acquisition [43]. Iwanow et al. [42] emphasise the role of empathic tendencies in shaping a positive attitude towards the acquisition of interpersonal skills. Nurses with empathic tendencies are capable of self-reflection, self-consciousness and constructive criticism of themselves, which can translate into shaping the need for a positive attitude towards acquiring social skills. Empathetic understanding, in addition to supporting positive attitudes towards learning social skills, also supports the process of shaping and using them. Empathic understanding allows nurses to understand patients’ emotions, attitudes and experiences when communicating with them. As a result, the nurse builds a better interpersonal relationship with the patient and can assess their actual needs and apply appropriate actions, offering services tailored to the patient [44]. Adopting the perspective of other people and understanding their emotional states determines the credibility of communication and promotes a better understanding of each other’s messages and their appropriate selection [45]. In addition, empathic understanding affects the sense of security in the interpersonal relationship with, and the information disclosed by, the patient, which significantly affects diagnosis, care planning and practice management [46]. Empathy also helps in non-verbal communication, allows for communication and the reading of emotional states, and breaks down prejudices towards the interlocutor [47]. Therefore, empathic understanding supports the development of social skills of nurses in a multifaceted way.

A person’s personality affects their attitude, feelings, beliefs and behaviour [48]. People with a type D personality are characterised by low self-esteem, a pessimistic attitude, chronic stress, a predisposition to burnout and less social contact. All these factors hinder the formation of social skills for such people and cause a sense of social isolation [32,49]. Cho et al. [50] reports that the percentage of type D personalities among nurses is higher than in the general population, ranging from 36% to 39%. This exposes D-type nurses to greater compassion fatigue, professional burnout, professional stress and PTSD than those without D-type personalities. In our study, 39% of PHC nurses were characterised as having a D-type personality, but it is worrying that already at the stage of their studies, this problem applies to nursing students. A study by Lee et al. [51] showed that the incidence of type D personality among nursing students is high, amounting to 33.5%. It is worth emphasising that the type D personality is not a temperament, but a modifiable personality that can be changed through intervention programs. In the studies by Nyklíček et al. [52], it was shown that the score for type D personality decreased in people who participated in an 8-week stress reduction program based on mindfulness. Personality explains social attitudes towards others and actions taken. It is an internal factor for the individual, which determines certain behaviours and often precedes social experiences. Depending on the personality type, nurses present different attitudes towards patients and the therapeutic team. Medical professions, in which a high level of altruism is demonstrated, are characterised by personality traits such as openness, agreeableness and conscientiousness [53]. Nurses with these personality types are open to interpersonal relationships, want to influence the working atmosphere, participate in meetings, are socially active, read non-verbal messages well and like to initiate and sustain a conversation. These features predispose them to a stronger tendency to engage in social training and thus contribute to better shaping of social skills [54,55].

In our research, we have proven that certain personal predispositions, such as empathic understanding and stress personality, are important predictors of social skills, so it is worth emphasising an individual approach during training and workshops on soft competences. Matczak [20] and Mroczek et al. [56] underline social skills training as a crucial determinant of having social skills. Social Skills Training (SST) can be delivered to a target population, for example, by conducting workshops and providing training. A person can also develop social competences in a natural environment through social interactions in real-life situations. During natural SST, social competences are developed through the trial-and-error method and by imitating social patterns. Every case that involves interaction with another person provides an opportunity for social training and the development of social competences [12,20,56]. Therefore, in addition to shaping social competences during their vocational training, each nurse has the opportunity to further develop their social competences in the course of natural social training throughout their professional career; the intensity of this training depends on many different factors. Social skills are a resource for both the organisation and the employee. Managers perceive social skills as one of the most important abilities in the work of nurses, which is why their formation both at the stage of studies, as well as in further stages of career development, is essential [57].

### Strengths and Limitations

The strengths of this research demand consideration. Firstly, to the best of our knowledge, this is the first study to evaluate empathic understanding and type D personality as correlates of social skills in a Polish cohort of nurses. Secondly, we made the best efforts to make the selection of our study group random, and the research was of a cross-cutting nature. Thirdly, we confirmed the need for a broader look at the problem of social skills among PHC nurses and the factors that determine them.

Our research has several limitations. Firstly, this study was conducted in one region of Poland, so it is difficult to extrapolate the results to the population of the whole country, or internationally. A study should be conducted amongst participants from different regions to confirm the results. Secondly, non-technical skills are perceived differently by different authors and consist of other skills. Selecting an appropriate tool to measure social skills was problematic, as there is no universal definition of social skills nor a nurse-specific assessment tool. A failure to agree on a model and a definition of social skills is an obstacle to assessing social skills and developing and implementing effective educational interventions. Thirdly, our study was based on closed-ended questionnaires, which may have narrowed the scope of the assessment. A qualitative approach, such as in-depth interviews or focus groups, is recommended to gather detailed information on social skills and discover new ideas for future research. Regardless of its limitations, this work brings several vital aspects that, to our knowledge, have not yet been addressed in the literature.

## 5. Conclusions

Our observations draw special attention to recognising the role of the individual resources of nurses in shaping their social skills, such as personality traits. A higher level of Empathic Understanding and not having a Type D personality were the predictors of higher levels of social skills.

Therefore, nurses’ social skills should be strengthened through the development and implementation of intervention programs containing Social Skills Training. However, it should be remembered that the individual characteristics of nurses need to be taken into account during training and workshops. For nurses with a Type D personality, it is advisable to additionally include some content in the training programs that would help reduce perceived stress.

Our research results ought to have some impact in creating a fuller perception of the possibilities of shaping the social skills of nursing staff and may constitute a recommendation for establishing Social Skills Training programs.

## Figures and Tables

**Table 1:** Distribution of analysed features in scales.

Scales	Results
Social Competences Profile (PROKOS)—Total score ^b^	172.27 ± 24.52
High ^a^	58 (13.00)
Average ^a^	291 (65.25)
Low ^a^	97 (21.75)
Subscale (A): Assertive competences—Total score ^b^	37.88 ± 6.44
High ^a^	61 (13.68)
Average ^a^	290 (65.02)
Low ^a^	95 (21.30)
Subscale (K): Cooperative competences—Total score ^b^	48.56 ± 6.56
High ^a^	41 (9.19)
Average ^a^	306 (68.61)
Low ^a^	99 (22.20)
Subscale (T): Social mindedness—Total score ^b^	30.82 ± 5.57
High ^a^	55 (12.33)
Average ^a^	306 (68.61)
Low ^a^	85 (19.06)
Subscale (Z): Social resourcefulness—Total score ^b^	38.84 ± 5.64
High ^a^	58 (13.01)
Average ^a^	295 (66.14)
Low ^a^	93 (20.85)
Subscale (S): Community awareness—Total score ^b^	16.17 ± 3.15
High ^a^	93 (20.85)
Average ^a^	322 (72.20)
Low ^a^	31 (6.95)
Empathic Understanding Questionnaire (KRE) ^b^	64.63 ± 10.16
Type D personality (DS-14)—Total score ^b^	20.24 ± 10.96
Negative Affectivity (NA) ^b^	11.28 ± 6.5
Social Inhibition (SI) ^b^	8.96 ± 5.38
Nurses with Type D personality ^a^	172 (39)

Note: Data presented as: ^a^ *n* (%) or ^b^ mean ± SD. Point ranges: PROKOS point range: 60–240, Subscale A point range: 14–56, Subscale K point range: 16–64, Subscale T point range: 11–44, Subscale Z point range: 13–52, S point range: 6–24, KRE point range: 0–99, DS-14 point range: 0–56, NA point range: 0–28, SI point range: 0–28.

**Table 2:** Relationship between empathy level assessment, type D personality and social skills.

Social Competences Profile (PROKOS)											
Total Score		Subscale A		Subscale K		Subscale T		Subscale Z		Subscale S	
b (SE)	*p*	b (SE)	*p*	b (SE)	*p*	b (SE)	*p*	b (SE)	*p*	b (SE)	*p*
Empathy level assessment:											
0.76 (0.11)	<0.001	0.14 (0.03)	<0.001	0.24 (0.03)	<0.001	0.14 (0.03)	<0.001	0.17 (0.03)	<0.001	0.07 (0.01)	<0.001
R^2^	15%		10%		20%		11%		13%		11%
Type D personality (reference category: No):											
−11.86 (2.28)	<0.001	−2.98 (0.61)	<0.001	−2.88 (0.61)	<0.001	−3.14 (0.52)	<0.001	−2.58 (0.53)	<0.001	−0.96 (0.3)	0.001
R^2^	11%		8%		10%		11%		9%		9%

Note: Multivariable model after adjustments for age, place of residence, postgraduate education and perceived health; Subscale A: Assertive competences; Subscale K: Cooperative competences; Subscale T: Social mindedness; Subscale Z: Social resourcefulness; Subscale S: Community awareness; b: standardised beta coefficient; SE: standard error; R^2^—coefficient of determination.

## Data Availability

The datasets generated during and/or analsed during the current study may be made available by the corresponding author on request.

## Supplementary Materials

The following supporting information can be downloaded at: https://www.mdpi.com/article/10.3390/ijerph20010201/s1, Table S1: STROBE Statement—Checklist; Table S2: Internal consistency of used questionnaires; Table S3: Descriptive statistics of surveyed respondents (*n* = 446); Table S4: Influence of selected variables on the level of social competences of the surveyed nurses; Table S5: Association between sociodemographic features, Empathic Understanding Questionnaire, Type D personality and subscales PROKOS Questionnaire—Part 1; Table S6: Association between sociodemographic features, Empathic Understanding Questionnaire, Type D personality and subscales PROKOS Questionnaire—Part 2.
