# Supplementary material for: Impact of the Empathic Understanding of People and Type D Personality as the Correlates of Social Skills of Primary Health Care Nurses: A Cross-Sectional Study
Source: Int J Environ Res Public Health. 2022 Dec 23;20(1):201. doi: 10.3390/ijerph20010201 (PMC9820031; doi:10.3390/ijerph20010201)
Supplement: Supplementary file 1 [file ijerph-20-00201-s001.zip › ijerph-2049837-supplementary.pdf]

International Journal of
Environmental Research and Public Health

MDPI

Supplementary materials

Impact of the Empathic Understanding of People and Type D Personality as the Correlates of Social Skills of Primary Health Care Nurses: A Cross-Sectional Study

Agnieszka Chrzan-Rodak[1], Grzegorz Józef Nowicki[1], Daria Schneider-Matyka[2*], ElżbietaGrochans[2] and Barbara Ślusarska[1]

Table S1. STROBE Statement—Checklist

	Item No	Recommendation	Page
**Title and abstract**	1	(*a*) Indicate the study's design with a commonly used term in the title or the abstract	1
		(*b*) Provide in the abstract an informative and balanced summary of what was done and what was found	1
**Introduction**			
Background/rationale	2	Explain the scientific background and rationale for the investigation being reported	1-4
Objectives	3	State specific objectives, including any prespecified hypotheses	4
**Methods**			
Study design	4	Present key elements of study design early in the paper	4-5
Setting	5	Describe the setting, locations, and relevant dates, including periods of recruitment, exposure, follow-up, and data collection	4-5
Participants	6	(*a*) Give the eligibility criteria, and the sources and methods of selection of participants	4-5
Variables	7	Clearly define all outcomes, exposures, predictors, potential confounders, and effect modifiers. Give diagnostic criteria, if applicable	N/A
Data sources/ measurement	8*	For each variable of interest, give sources of data and details of methods of assessment (measurement). Describe comparability of assessment methods if there is more than one group	N/A
Bias	9	Describe any efforts to address potential sources of bias	4-5
Study size	10	Explain how the study size was arrived at	4-5

Quantitative variables	11	Explain how quantitative variables were handled in the analyses. If applicable, describe which groupings were chosen and why	N/A
Statistical methods	12	(*a*) Describe all statistical methods, including those used to control for confounding	7-8
		(*b*) Describe any methods used to examine subgroups and interactions	7-8
		(*c*) Explain how missing data were addressed	4-5
		(*d*) If applicable, describe analytical methods taking account of sampling strategy	N/A
		(*e*) Describe any sensitivity analyses	N/A
**Results**			
Participants	13*	(a) Report numbers of individuals at each stage of study—eg numbers potentially eligible, examined for eligibility, confirmed eligible, included in the study, completing follow-up, and analysed	4-5
		(b) Give reasons for non-participation at each stage	4-5
		(c) Consider use of a flow diagram	4-5
Descriptive data	14*	(a) Give characteristics of study participants (eg demographic, clinical, social) and information on exposures and potential confounders	8
		(b) Indicate number of participants with missing data for each variable of interest	N/A
Outcome data	15*	Report numbers of outcome events or summary measures	N/A
Main results	16	(*a*) Give unadjusted estimates and, if applicable, confounder-adjusted estimates and their precision (eg, 95% confidence interval). Make clear which confounders were adjusted for and why they were included	N/A
		(*b*) Report category boundaries when continuous variables were categorized	N/A
		(*c*) If relevant, consider translating estimates of relative risk into absolute risk for a meaningful time period	N/A
Other analyses	17	Report other analyses done—eg analyses of subgroups and interactions, and sensitivity analyses	N/A
**Discussion**			
Key results	18	Summarise key results with reference to study objectives	11-12
Limitations	19	Discuss limitations of the study, taking into account sources of potential bias or imprecision. Discuss both direction and magnitude of any potential bias	13-14

Interpretation	20	Give a cautious overall interpretation of results considering objectives, limitations, multiplicity of analyses, results from similar studies, and other relevant evidence	11-13
Generalisability	21	Discuss the generalisability (external validity) of the study results	11-13
**Other information**			
Funding	22	Give the source of funding and the role of the funders for the present study and, if applicable, for the original study on which the present article is based	15

*Give information separately for exposed and unexposed groups.

Note: An Explanation and Elaboration article discusses each checklist item and gives methodological background and published examples of transparent reporting. The STROBE checklist is best used in conjunction with this article (freely available on the Web sites of PLoS Medicine at http://www.plosmedicine.org/, Annals of Internal Medicine at http://www.annals.org/, and Epidemiology at http://www.epidem.com/). Information on the STROBE Initiative is available at www.strobe-statement.org.

Table S2. Internal consistency of used questionnaires

Questionnaire	*alfa* Cronbach
Social Competencies Profile (PROKOS) [1]	0.92
Subscale (A): Assertive competences	0.90
Subscale (K): Cooperative competences	0.90
Subscale (T): Social mindedness	0.89
Subscale (Z): Social resourcefulness	0.88
Subscale (S): Community awareness	0.80
Empathic Understanding Questionnaire (KRE) [2]	0.85
Type D personality (DS-14)	0.92
Negative Affectivity (NA)	0.90
Social Inhibition (SI)	0.85

[1]The symbols used to designate subscales in the PROKOS questionnaire are derived from the Polish names of these subscales. We have left the abbreviations of the original symbols because the English version of this tool is missing, and the authors of the questionnaire have not yet published the names of these subscales in a language other than the original one.
[2]We left the original abbreviation of the KRE scale, because the English version of this tool is missing, and in the literature, the authors often use the abbreviation KRE.

Table S3. Descriptive statistics of surveyed respondents (n = 446)

Characteristic	Group	Study Group
Age (years) [b]		47 ± 10.19
Work experience (years) [b]		24 ± 11.53
Gender (female) [a]		401 (89.91)
Marital status (in a relationship) [a]		373 (83.63)
Place of residence [a]	Village (≤ 8000 residents)	141 (31.61)
	Town (< 100 000 residents)	206 (46.19)
	City (> 100 000 residents)	99 (22.20)
Education [a]	Registered nurse (secondary nursing school - lyceum) [1]	168 (37.67)
	Registered nurse with bachelor's degree in nursing	156 (34.98)
	Registered nurse with master's degree in nursing	122 (27.35)
Postgraduate education (yes) [a]		390 (87.44)
	Specialization	137 (34.95)
	Qualification course	305 (77.81)
	Specialist course	270 (68.88)
Postgraduate education in the last 2 years (yes) [a]		299 (67.04)
Additional job (yes) [a]		140 (31.39)
Perceived health [b]		3.08 ± 0.69

Note: Data presented as: [a]n (%) or [b]mean ± SD; [1]Secondary Nursing School (Lyceum)—Nursing education system in Poland before 1999.

Table S4. Influence of selected variables on the level of social competences of the surveyed nurses

**Variable**	**Social Competencies Profile (PROKOS)**											
	Total score	*p*	Subscale A	*p*	Subscale K	*p*	Subscale T	*p*	Subscale Z	*p*	Subscale S	*p*
Age [year] [a]	-0.097	0.04	-0.082	0.08	-0.058	0.22	-0.120	0.01	-0.097	0.04	-0.083	0.08
Gender [b]												
Famale	172.8 ± 24.51	0.18	38.02 ± 6.45	0.17	48.71 ± 6.52	0.16	30.86 ± 5.61	0.66	38.99 ± 5.61	0.10	16.22 ± 3.13	0.43
Male	167.58 ± 24.36		36.64 ± 6.29		47.2 ± 6.82		30.49 ± 5.18		37.44 ± 5.82		15.8 ± 3.31	
Marital status [b]												
Single	171.08 ± 27.21	0.68	37.81 ± 7.25	0.92	47.73 ± 6.98	0.26	31.33 ± 5.81	0.41	37.97 ± 6.39	0.20	16.25 ± 3.47	0.85
In relationship	172.51 ± 23.99		37.90 ± 6.28		48.72 ± 6.47		30.72 ± 5.52		39.01 ± 5.48		16.16 ± 3.09	
Place of residence [c]												
Village (≤ 8000 residents)	171.64 ± 22.69	0.12	37.47 ± 6.01	0.15	48.54 ± 6.54	0.24	30.87 ± 5.06	0.10	38.78 ± 5.55	0.35	15.99 ± 2.69	0.04
Town (< 100 000 residents)	170.59 ± 25.25		37.63 ± 6.54		48.13 ± 6.67		30.33 ± 5.71		38.54 ± 5.66		15.96 ± 3.32	
City (> 100 000 residents)	176.69 ± 25.18		38.99 ± 6.74		49.48 ± 6.31		31.79 ± 5.87		39.54 ± 5.74		16.89 ± 3.31	
Education [c]												
Nurse after Secondary Nursing School	171.38 ± 21.61	0.73	37.39 ± 5.95	0.34	48.56 ± 5.68	0.99	30.61 ± 5.01	0.71	38.76 ± 5.06	0.96	16.06 ± 2.97	0.51
Nurse with Bachelor's Degree	172.12 ± 27.66		37.91 ± 6.93		48.52 ± 7.52		30.78 ± 6.27		38.83 ± 6.16		16.08 ± 3.29	
Nurse with Master's Degree	173.70 ± 24.14		38.52 ± 6.42		48.61 ± 6.41		31.16 ± 5.36		38.96 ± 5.76		16.46 ± 3.21	
Postgraduate education [b]												
Yes	171.82 ± 23.88	0.37	37.83 ± 6.44	0.68	48.50 ± 6.32	0.70	30.57 ± 5.41	0.03	38.70 ± 5.51	0.24	16.21 ± 3.07	0.59
No	175.43 ± 28.63		38.21 ± 6.48		48.95 ± 8.09		32.55 ± 6.35		39.79 ± 6.47		15.93 ± 3.66	
Postgraduate education in the last 2 years [b]												
Yes	174.04 ± 24.61	0.03	38.33 ± 6.53	0.03	49.07 ± 6.48	0.02	30.97 ± 5.55	0.41	39.19 ± 5.66	0.06	16.48 ± 3.17	0.003
No	168.67 ± 24.01		36.97 ± 6.17		47.51 ± 6.60		30.51 ± 5.61		38.12 ± 5.55		15.56 ± 3.02	
Additional job [b]												
Yes	172.45 ± 27.39	0.92	38.22 ± 7.19	0.46	48.17 ± 7.16	0.42	31.20 ± 5.99	0.35	38.44 ± 5.97	0.33	16.41 ± 3.45	0.30
No	172.17 ± 23.17		37.71 ± 6.07		48.73 ± 6.28		30.65 ± 5.37		39.02 ± 5.5		16.06 ± 3.0	
Perceived health [a]	0.180	< 0.001	0.171	<0.001	0.186	<0.001	0.131	0.005	0.145	0.002	0.168	<0.001
Empathic Understanding Questionnaire (EUQ) [a]												
Total score	0.283	<0.001	0.191	<0.001	0.346	<0.001	0.234	<0.001	0.275	<0.001	0.186	<0.001
Type D personality (DS-14) [b]												
Nurses with Type D personality	164.51 ± 22.02	<0.001	36.35 ± 6.19	<0.001	46.68 ± 5.70	<0.001	28.80 ± 4.88	<0.001	37.16 ± 5.23	<0.001	15.52 ± 2.99	<0.001
Nurses without Type D personality	177.15 ± 24.78		38.84 ± 6.41		49.74 ±6.79		32.09 ± 5.60		39.89 ± 5.65		16.59 ± 3.18	

Note: [a] r - Pearson's correlation coefficient; [b] t-Student test; [c] ANOVA; Subscale A: Assertive competences; Subscale K: Cooperative competences; Subscale T: Social mindedness; Subscale Z: Social resourcefulness; Subscale S: Community awareness

Table S5. Association between sociodemographic features, Empathic Understanding Questionnaire, Type D personality and subscales PROKOS Questionnaire- Part 1

Variable	Total						Subscale A						Subscale K					
	b (SE)	*p*	b (SE)	*p*	b (SE)	*p*	b (SE)	*p*	b (SE)	*p*	b (SE)	*p*	b (SE)	*p*	b (SE)	*P*	b (SE)	*p*
Age	-0.12 (0.12)	0.31	-0.23 (0.11)	0.037	-0.09 (0.11)	0.415	-0.02 (0.03)	0.425	-0.05 (0.03)	0.135	-0.02 (0.03)	0.52	0.003 (0.03)	0.913	-0.04 (0.03)	0.169	0.003 (0.03)	0.925
Town (reference category: Village)	-0.42 (2.65)	0.875	-0.71 (2.51)	0.777	-0.51 (2.57)	0.844	0.31 (0.7)	0.659	0.25 (0.68)	0.709	0.29 (0.69)	0.673	-0.32 (0.71)	0.65	-0.42 (0.66)	0.527	-0.34 (0.69)	0.62
City (reference category: Village)	4.99 (3.15)	0.113	4.82 (2.99)	0.107	4.36 (3.06)	0.155	1.51 (0.83)	0.069	1.48 (0.81)	0.068	1.39 (0.82)	0.09	0.94 (0.84)	0.266	0.88 (0.78)	0.258	0.79 (0.82)	0.341
Postgraduate education in the last 2 years (reference category: No)	-4.84 (2.43)	0.047	-5.9 (2.31)	0.011	-4.76 (2.36)	0.045	-1.23 (0.64)	0.056	-1.42 (0.63)	0.024	-1.21 (0.63)	0.056	-1.48 (0.65)	0.024	-1.81 (0.6)	0.003	-1.46 (0.64)	0.022
Perceived health	5.85 (1.68)	<0.001	5.84 (1.59)	<0.001	5.47 (1.63)	<0.001	1.48 (0.44)	<0.001	1.48 (0.43)	<0.001	1.41 (0.44)	0.001	1.7 (0.45)	<0.001	1.7 (0.42)	<0.001	1.61 (0.44)	<0.001
Empathy level assessment			0.76 (0.11)	<0.001					0.14 (0.03)	<0.001					0.24 (0.03)	<0.001		
Type D personality (reference category: No):					-11.86 (2.28)	<0.001					-2.98 (0.61)	<0.001					-2.88 (0.61)	<0.001
R^2		6%		15%		11%		5%		10%		8%		6%		20%		10%

Table S6. Association between sociodemographic features, Empathic Understanding Questionnaire, Type D personality and subscales PROKOS Questionnaire- Part 2

Variable	Subscale T						Subscale Z						Subscale S					
	b (SE)	*p*	b (SE)	*p*	b (SE)	*p*	b (SE)	*p*	b (SE)	*p*	b (SE)	*p*	b (SE)	*p*	b (SE)	*p*	b (SE)	*p*
Age	-0.05 (0.03)	0,076	-0.07 (0.03)	0,008	-0.04 (0.03)	0.115	-0.03 (0.03)	0.221	-0.06 (0.03)	0.024	-0.03 (0.03)	0.298	-0.01 (0.01)	0.503	-0.02 (0.01)	0.173	-0.01 (0.01)	0.594
Town (reference category: Village)	-0.35 (0.61)	0.567	-0.4 (0.59)	0.491	-0.37 (0.58)	0.525	-0.09 (0.61)	0.886	-0.15 (0.59)	0.793	-0.11 (0.6)	0.858	0.03 (0.34)	0.921	0.01 (0.33)	0.981	0.03 (0.33)	0.937
City (reference category: Village)	0.91 (0.72)	0.208	0.88 (0.7)	0.209	0.74 (0.69)	0.286	0.74 (0.73)	0.311	0.7 (0.7)	0.313	0.6 (0.71)	0.397	0.9 (0.4)	0.026	0.88 (0.39)	0.026	0.84 (0.4)	0.035
Postgraduate education in the last 2 years (reference category: No)	-0.33 (0.56)	0.558	-0.53 (0.54)	0.327	-0.3 (0.54)	0.57	-0.95 (0.56)	0.094	-1.18 (0.54)	0.029	-0.93 (0.55)	0.092	-0.87 (0.31)	0.006	-0.96 (0.3)	0.002	-0.86 (0.31)	0.005
Perceived health	0.9 (0.38)	0.02	0.9 (0.37)	0.016	0.8 (0.37)	0.032	1.06 (0.39)	0.007	1.06 (0.37)	0.005	0.98 (0.38)	0.011	0.71 (0.21)	0.001	0.71 (0.21)	0.001	0.68 (0.21)	0.002
Empathy level assessment			0.14 (0.03)	<0.001					0.17 (0.03)	<0.001					0.07 (0.01)	<0.001		
Type D personality (reference category: No):					-3.14 (0.52)	<0.001					-2.58 (0.53)	<0.001					-0.96 (0.3)	0.001
R^2		4%		11%		11%		5%		13%		9%		6%		11%		9%

Note: Subscale A: Assertive competences; Subscale K: Cooperative competences; Subscale T: Social mindedness; Subscale Z: Social resourcefulness; Subscale S: Community awareness; b: standardized beta coefficient; SE: standard error; R^2 -coefficient of determination.
